# Retroareolar Carcinomas in Breast Ultrasound: Pearls and Pitfalls

**DOI:** 10.3390/cancers9010001

**Published:** 2016-12-30

**Authors:** Romuald Ferré, Martine Paré, Lisa Smith, Mélanie Thériault, Ann Aldis, Ellen Kao, Benoit Mesurolle

**Affiliations:** 1 Radiology Department, North Ontario School of Medicine, Thunder Bay Regional Health Centre, Thunder Bay, ON P7B 6V4, Canada; 2 Department of Radiology, McGill University Health Center, Royal Victoria Hospital, 687 Pine Ave West, Montreal, QC H3H 1A1, Canada; martinepare@videotron.ca (M.P.); lissmi28@yahoo.com (L.S.); melanie.theriault@videotron.ca (M.T.); aealdis@hotmail.com (A.A.); ellen.kao@muhc.mcgill.ca (E.K.); bmesurolle@yahoo.fr (B.M.)

**Keywords:** breast, retroareolar, carcinomas, ultrasound

## Abstract

Breast Ultrasound (US) is an important tool for both screening and diagnostic examinations. Although breast US has benefitted from significant recent technical improvements, its use for the retroareolar region is known to be more challenging than for other locations. The retroareolar location was defined by Giess et al. in 1998 as the region where any lesion is situated at less than two cm from the nipple and/or involves the nipple-areolar complex on mammogram. Understanding of the complex anatomy and physiology of the nipple-areolar region is important to avoid misinterpretation and misdiagnosis. The ability for the breast imager to manage difficulties related to the retroareolar area is paramount by adjusting settings (compounding, frequency, Doppler) and utilizing specific manoeuvers. Cases illustrating difficulties encountered in diagnosis of retroareolar carcinomas are presented.

## 1. Introduction

**Figure 1:**
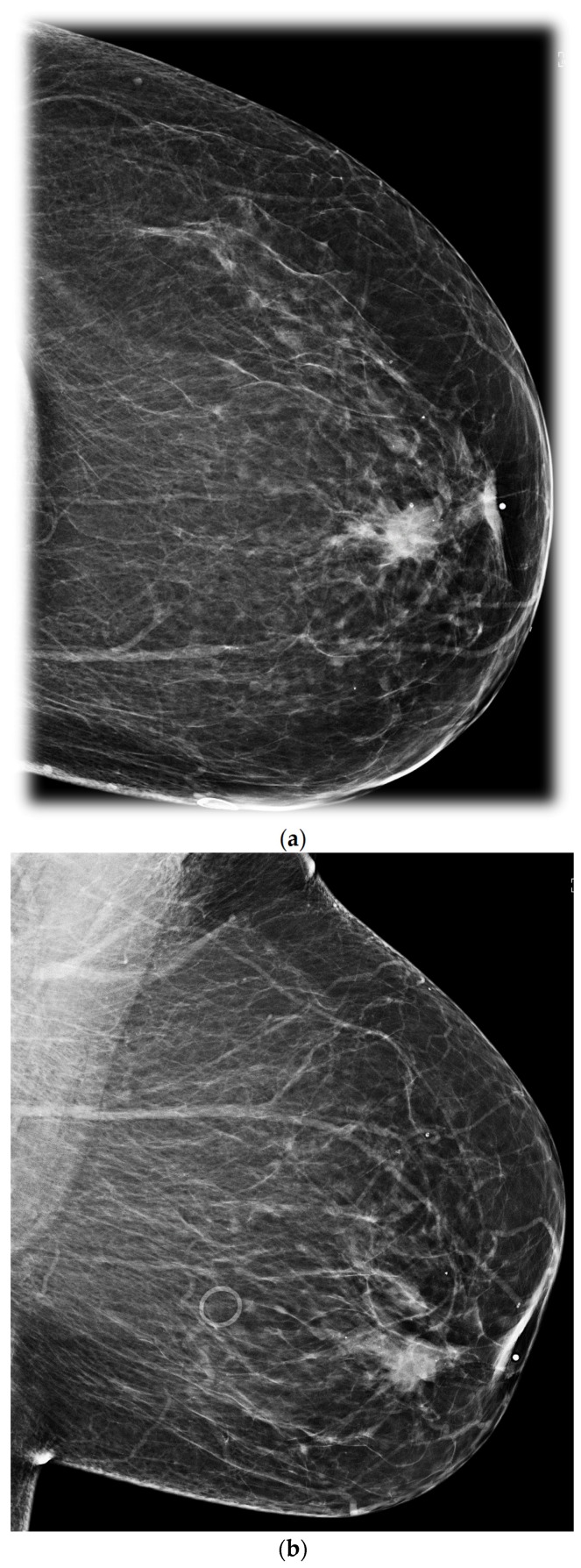
Retroareolar carcinoma. CC (**a**) and MLO (**b**) mammograms show a spiculated irregular mass containing intralesional calcifications. The mass measures 17 mm and is located in the retroareolar region at less than 2 cm from the nipple and associated with a nipple inversion.

The retroareolar region is considered as a region with special characteristics and challenges in breast diseases [1,2,3,4]. Although the nipple is an important anatomic landmark routinely used for localization in breast ultrasound (US) by measuring the lesion-to-nipple distance, the literature does not provide a clear definition of the retroareolar lesion in this modality [5]. Giess et al. proposed a definition of retroareolar lesions based on mammographic criteria. A lesion is deemed retroareolar if located within two centimeters from the nipple-areolar complex [5] (Figure 1).

Breast carcinomas situated in the retroareolar region account for 8% of breast cancers and are considered more difficult to diagnose than cancers elsewhere in the breast [3,6,7,8,9]. Contrary to the clinical examination that is considered sensitive for detection of retroareolar masses, mammography and US can easily miss them [3,5]. Despite continuous and significant improvement in breast US with the development of high frequency transducers, compound imaging and speckle reduction algorithms [4,10,11,12,13], scanning the retroareolar location with US is still challenging and can be affected by many artifacts [14].

A review of the anatomy and the factors contributing to the difficulties in scanning this region with ultrasound is presented, followed with some tips to optimize imaging techniques, relevant pathologic findings and demonstration of some pitfalls.

## 2. Anatomy

The retroareolar region is situated behind the nipple-areolar complex, a major landmark in the breast, specialized in collecting and expressing breast milk during lactation [6]. The nipple-areolar complex contains essentially Montgomery glands opening at Morgagni tubercles, smooth muscle, nerves sensory endings and Sappey plexus, the retroareolar lymphatic system [1].

## 3. US Imaging Modalities

### 3.1. US Technical Challenges

**Figure 2:**
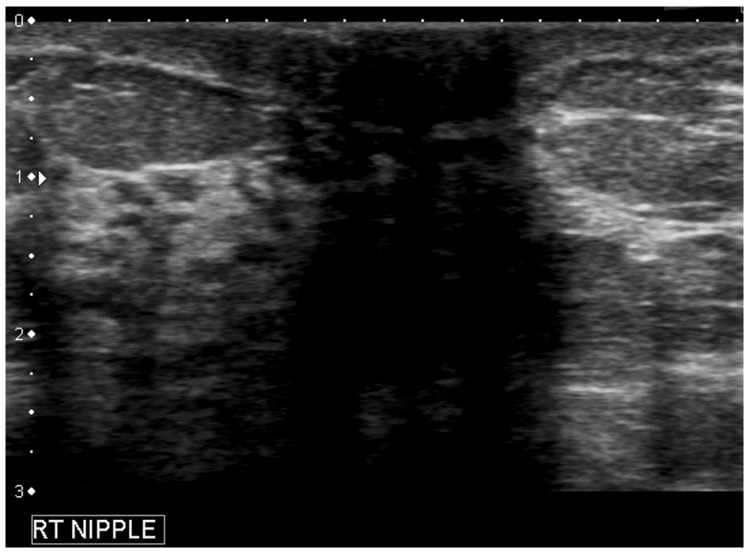
US image of the retroareolar area in a 64 year-old patient. Acoustic shadowing due to the nipple. Crevices and irregular surfaces can be responsible for a mass-like appearance.

**Figure 3:**
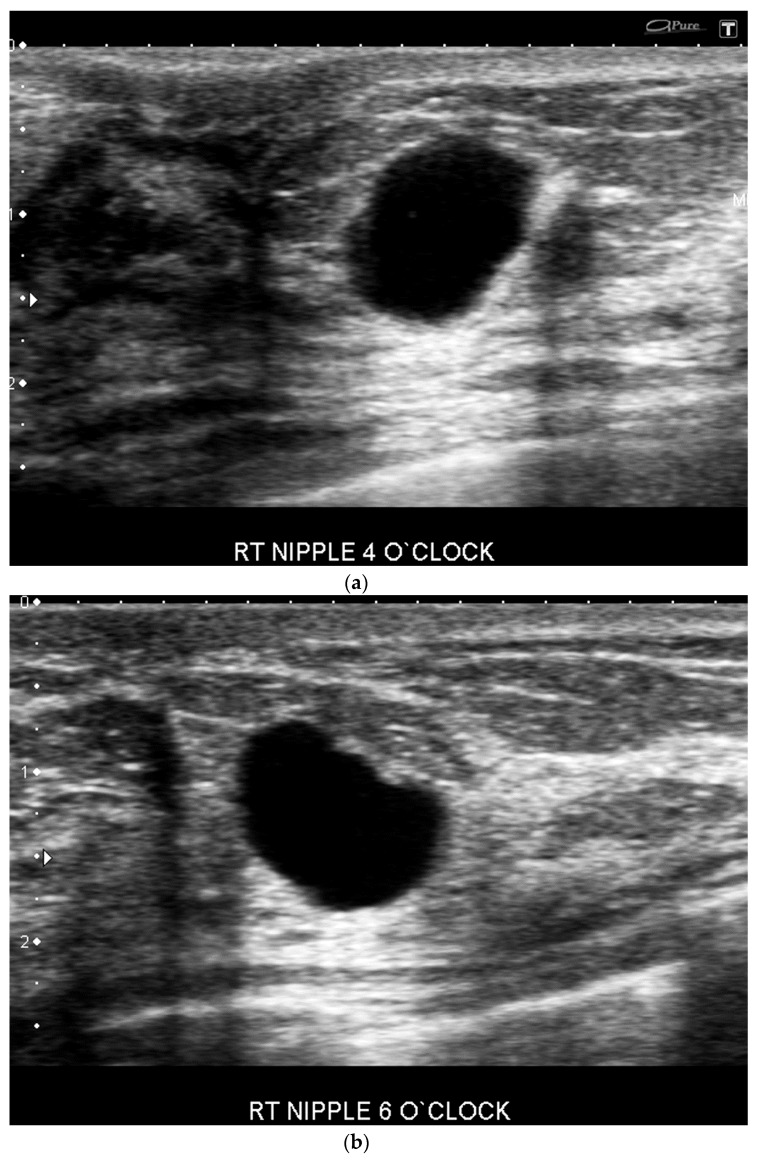
Different locations of a same retroareolar lesion depending on the breast imager. A retroareolar simple cyst is labeled sonographically at 4 o’clock by one breast imager (**a**) and 6 o’clock (**b**) by another one.

**Figure 4:**
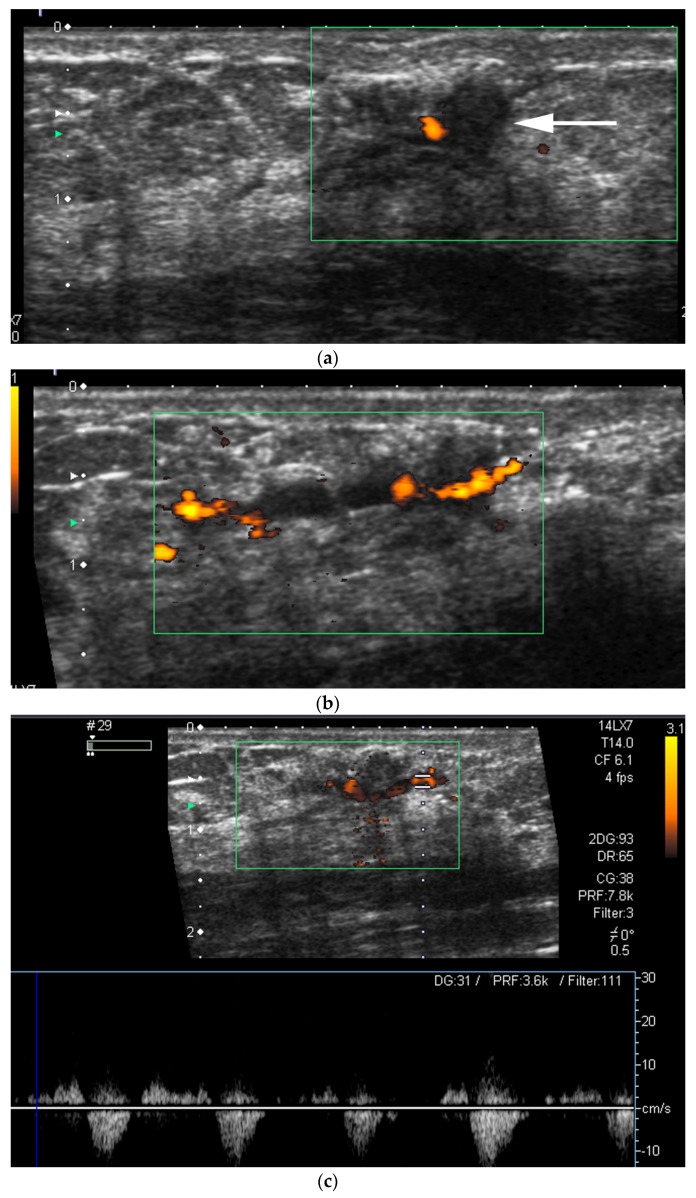
US-guided procedures images of a clinically palpable retroareolar mass in a 81 year-old patient on aspirin. Transverse US images show a hypoechoic mass with angular margins measuring 6 mm associated with a rim vascularity at color Doppler interrogation (**a**) (arrow). Sonographically guided 14-gauge core biopsy. Post biopsy US image shows an active bleeding at the biopsy site (**b**,**c**). Pathology yielded intermediate-grade invasive ductal carcinoma.

**Figure 5:**
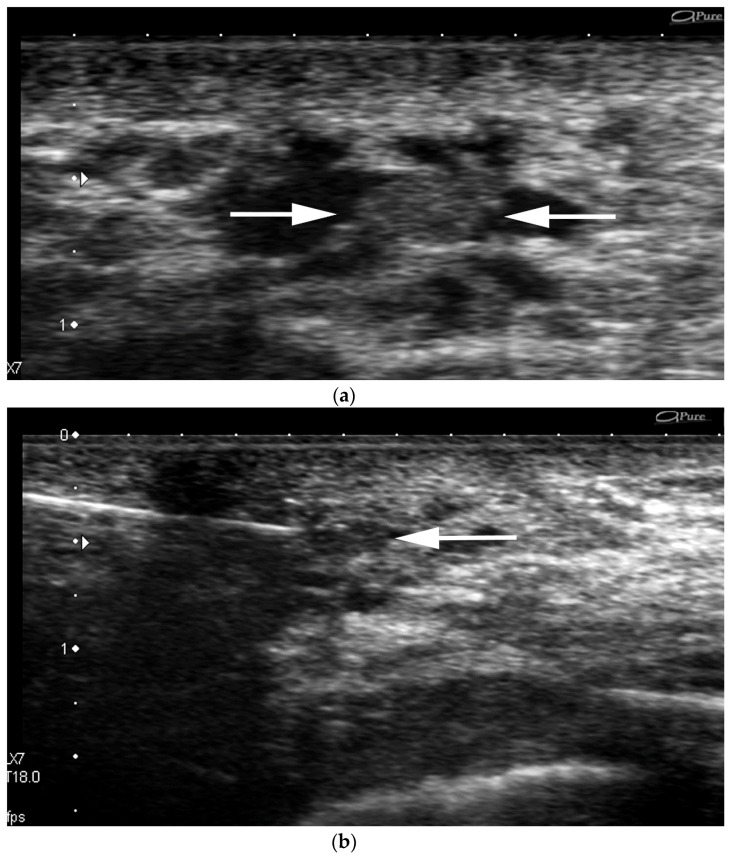
US-guided procedures images of a retroareolar lesion. Evidence of a new retroareolar microlobulated isoechoic mass measuring 4 mm within a dilated duct in a 57 year-old patient (**a**) (arrows). The intraductal lesion becomes less visible after freezing (**b**) (arrow). US-guided per procedure image of the same structure. The lesion is no longer visualized after one pass. Pathology yielded papilloma.

Several factors can explain why ultrasound of the retroareolar region can be challenging:

- Acoustic shadowing is the main culprit and is related to two simultaneous factors:
  ○ Geometric shape of the nipple: the crevices and irregular surfaces may generate a mass like appearance with posterior acoustic shadowing (Figure 2).
  ○ Ducts have a radial orientation that limits US evaluation given the beam direction.
- Increased inter-observer variation in labelling the location. In our practice, we noted that a lesion adjacent to the nipple can be differently labeled with respect to location by different operators Depending on the probe position related to the nipple, the clock-face location can vary (e.g., the same retroareolar lesion can be labelled at 12 o’clock by one imager and 6 o’clock by a second one) (Figure 3).
- US-guided procedures in the nipple-areolar region can be more complex, due to the increased sensitivity and vascularity of the area (Figure 4). The presence of shadowing, and the abundant amount of anesthetic necessary for the procedure can mask the targeted lesion. In addition, an intraductal lesion can become less visible after injection of local anesthetics (especially when injected into the ducts) and after multiple biopsy passes, when the cystic component collapses. Therefore, the sampling risks being less accurate (Figure 5).

### 3.2. Tips Proposed to Improve US Scanning of Retroareolar Structures

**Figure 6:**
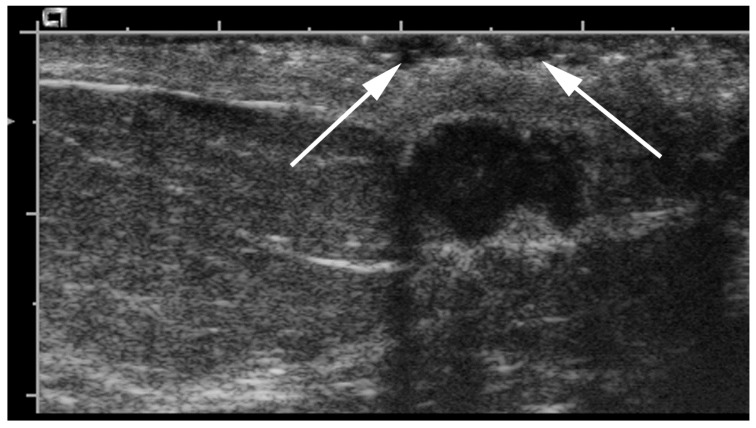
US images of a highly suspicious retroareolar mass after a thick pad of gel in a 56-year-old patient (arrows). US-guided core needle biopsy demonstrated invasive ductal carcinoma.

**Figure 7:**
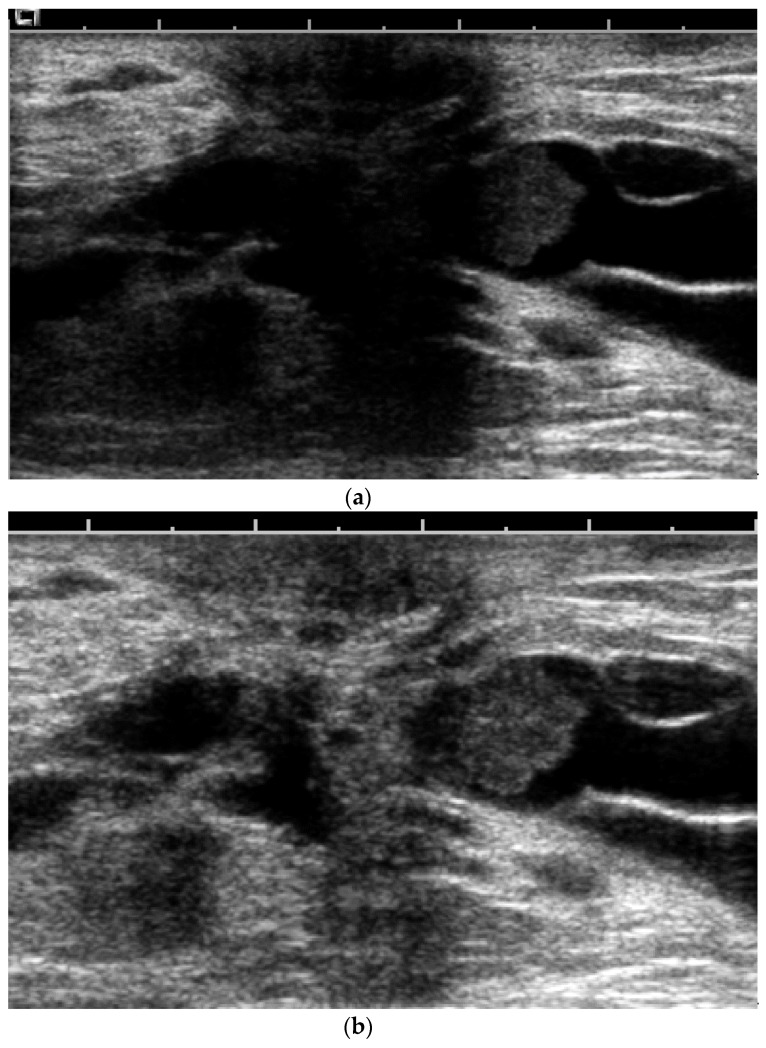
US image of an intra-ductal papilloma. Image (**a**), using Tissue Harmonic Imaging shows evidence of posterior acoustic shadowing generated by the nipple. Image (**b**) shows that the posterior shadowing significantly decreases with use of frequency compounding imaging, with a better visibility of the retroareolar region.

**Figure 8:**
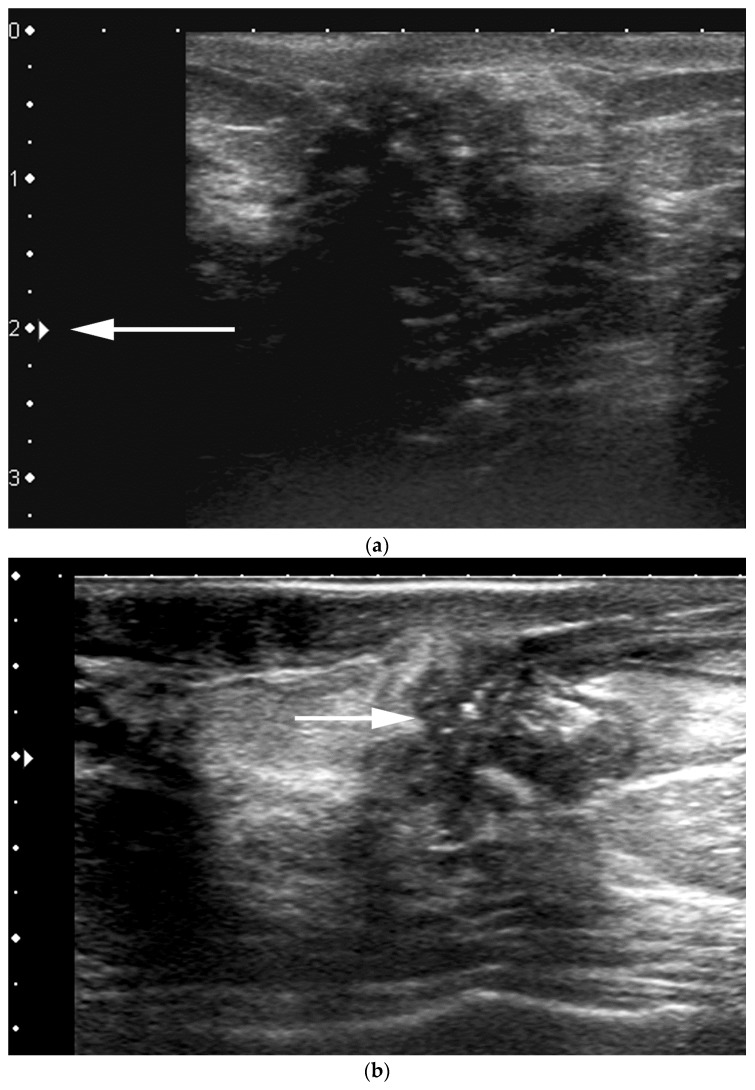
US images of a suspicious retroareolar mass in 64-year-old patient. Focal position is not accurate (**a**) (arrow). An optimal focal adjustement (**b**) allows an improved visualization of the suspicious mass. US-guided core needle demonstrated an invasive ductal carcinoma.

**Figure 9:**
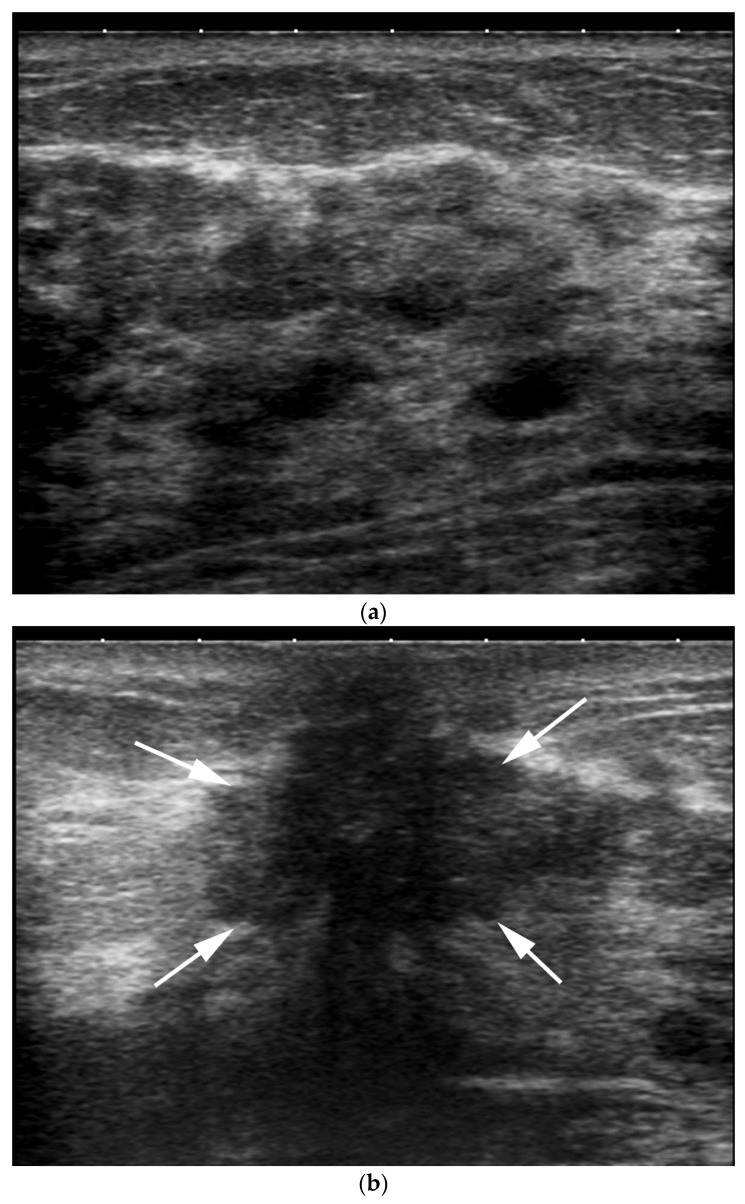
Comparison with the contralateral side can help the breast imager. US transverse images of the right (**a**) and left (**b**) retroareolar area. Right retroareolar area shows a simple cyst (**a**) versus left retroareolar area (**b**) shows a subtle hypoechoic irregular mass with posterior shadowing (arrows). Pathologic analysis yielded invasive ductal carcinoma.

- A thick pad of gel can aid the operator to compensate for the geometric shape of the nipple. The abundant gel replaces the air trapped in the crevices and decreases the artifacts. The gel also allows the visualization of the more superficial structures (Figure 6).
- Optimizing settings of the ultrasound machine helps reducing artifacts as well. Spatial and frequency compounding decreases the shadowing compared to Tissue Harmonic imaging or fundamental imaging [10,11] (Figure 7). An adjusted focal zone is also part of optimizing settings (Figure 8).
- Comparison with the contralateral side is helpful, especially in case of subtle retroareolar findings (Figure 9).

### 3.3. Proper Scanning Techniques

Stavros described several maneuvers that may improve the detection and characterization of retro-areolar lesions. These techniques are based on angulation of the transducer in order to generate an ultrasound beam perpendicular to the long axis of the duct [14,15,16,17].

**Figure 10:**
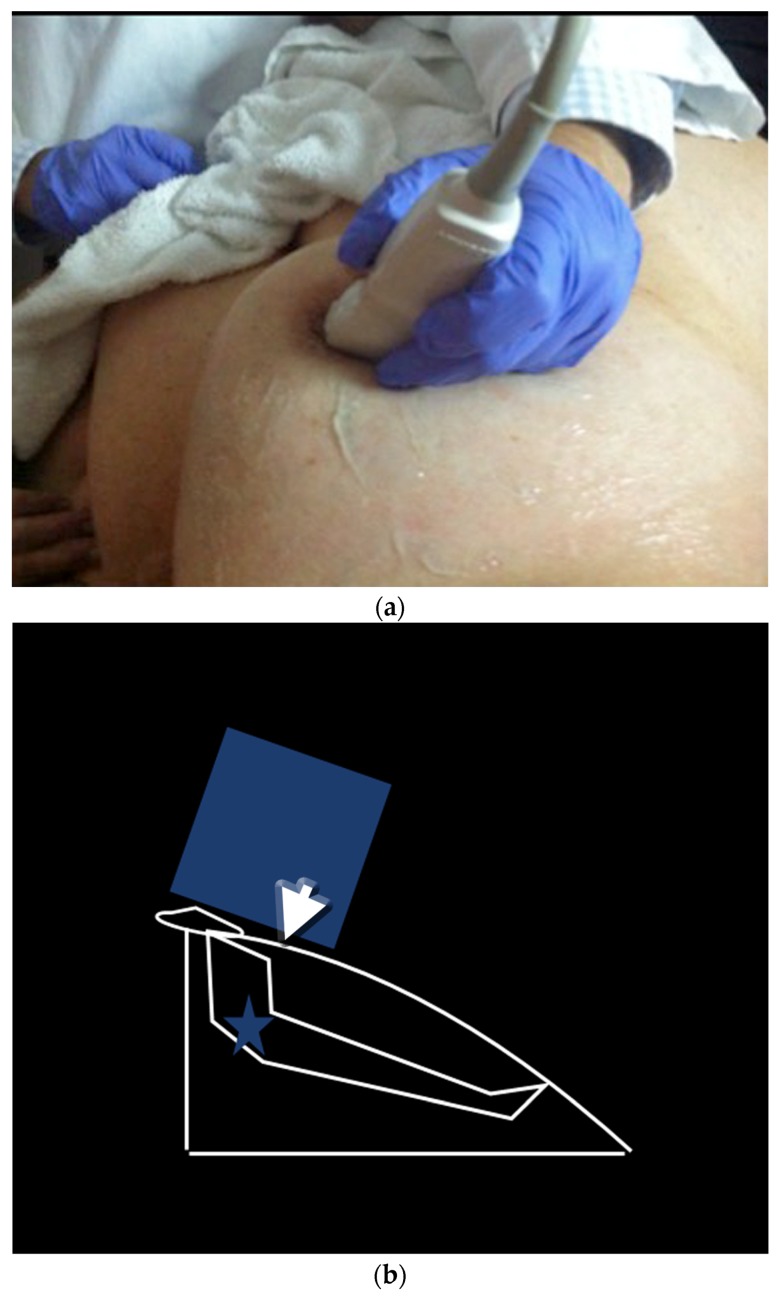

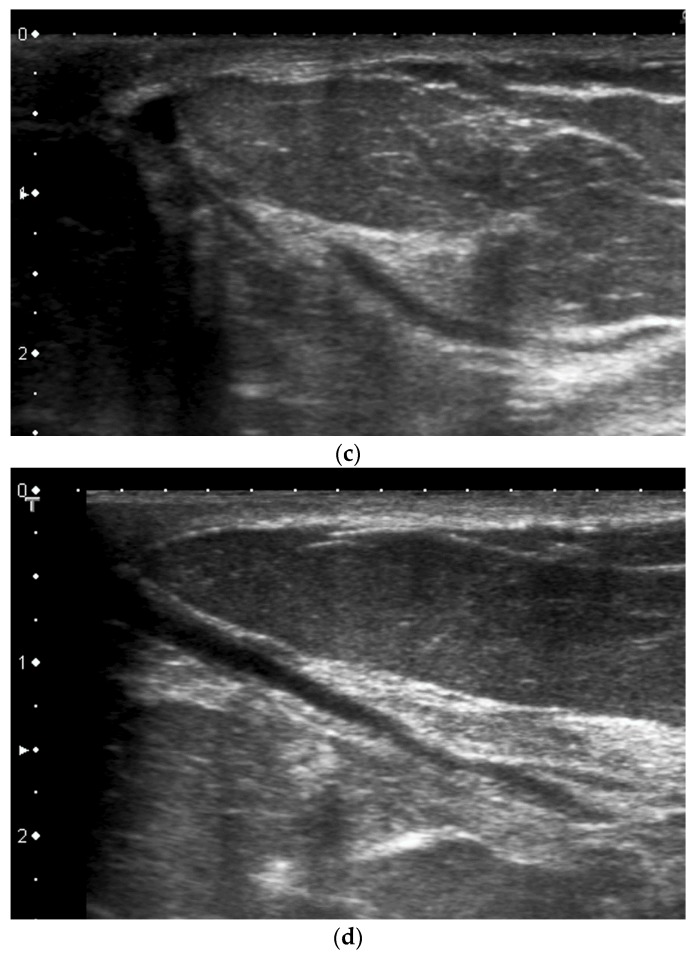
Peripheral compression technique can aid to visualize the peripheral segments of retroareolar ducts. Photograph of the manoeuver (**a**) and its corresponding drawing (**b**). US pre manoeuver (**c**) and post manoeuver (**d**) images.

**Figure 11:**
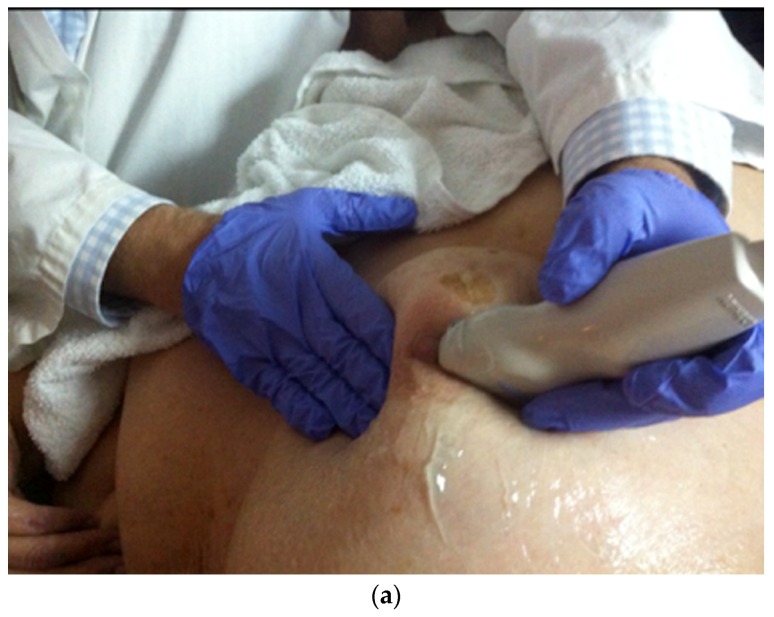

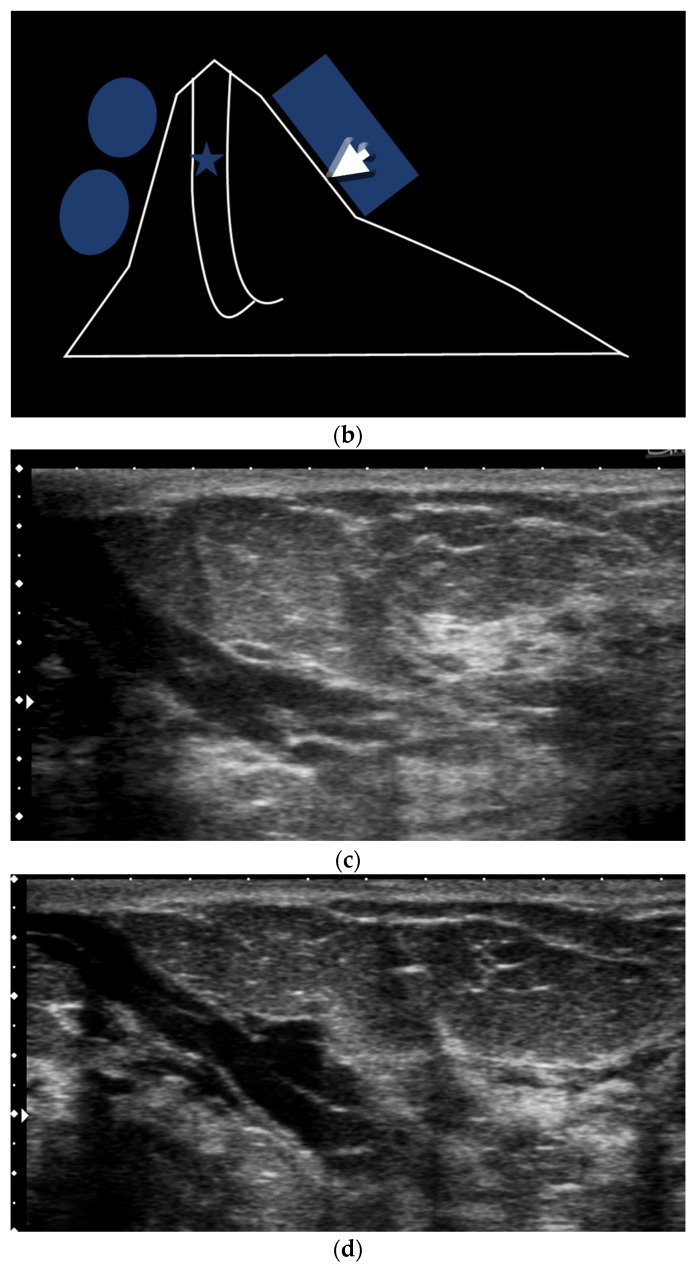
Two-handed compression technique compresses the retroareolar duct between the non-scanning hand and the probe. Photograph of the manoeuver (**a**) and its corresponding drawing (**b**). US pre manoeuver (**c**) and post manoeuver (**d**) images.

**Figure 12:**
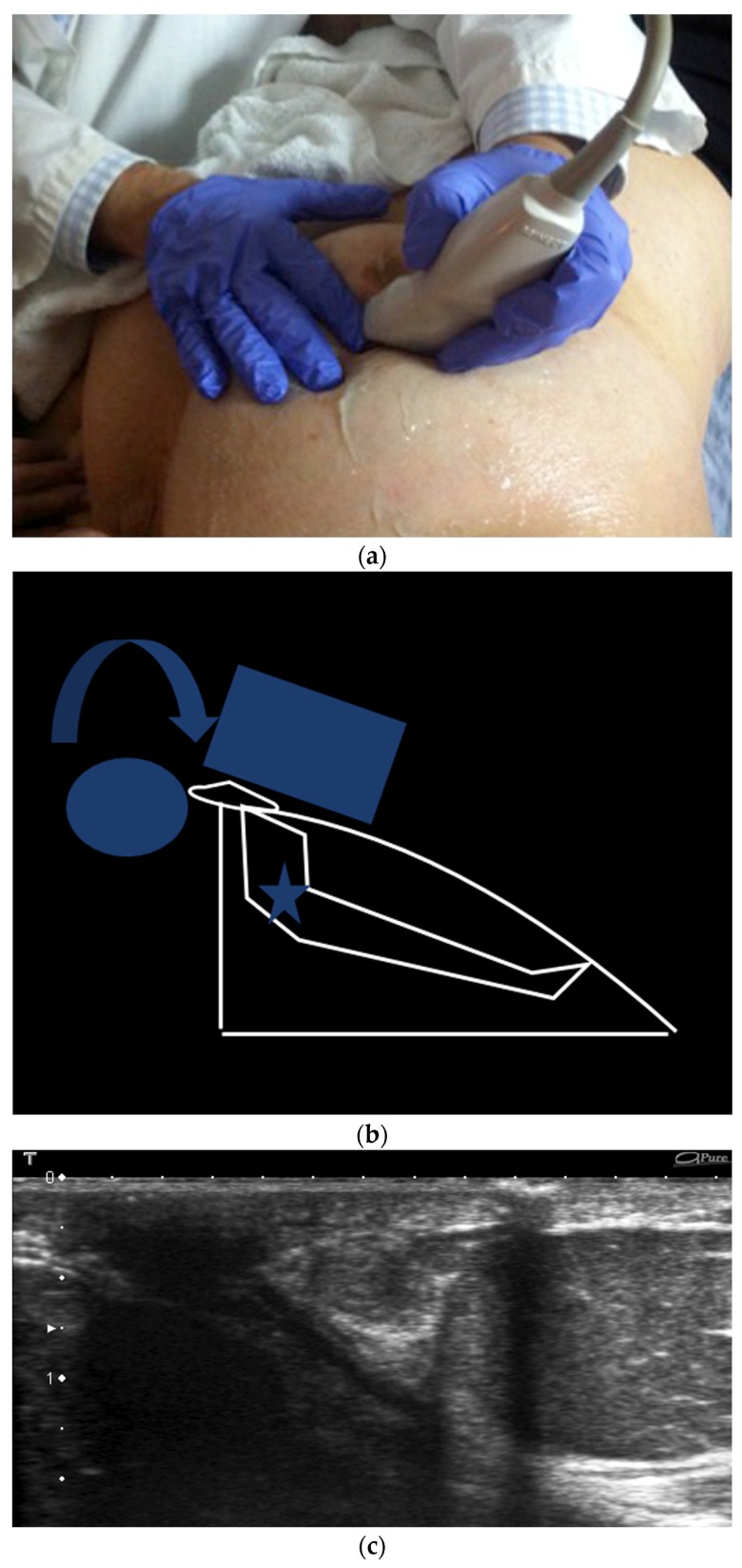

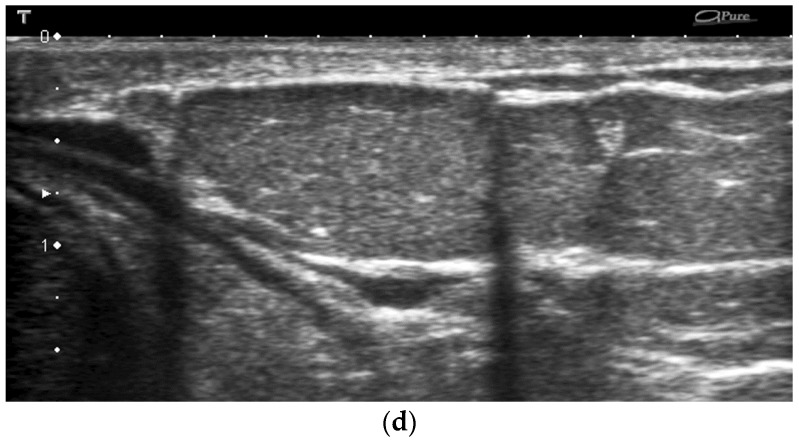
Rolled nipple technique. The nipple is rolled towards the finger of the contralateral hand with the probe. Photograph of the manoeuver (**a**) and its corresponding drawing (**b**). US pre manoeuver (**c**) and post manoeuver (**d**) images.

- The peripheral compression technique (Figure 10)
  ○ For visualization of the peripheral retroareolar duct segments
  ○ Performed with a nipple compression on the lateral end of the probe. The tranducer is held with an angle. The beam is then perpendicular to the duct and simultaneously the probe maintains contact and pressure.
- The two-handed compression technique (Figure 11)
  ○ For visualization of the central retroareolar duct segments
  ○ The two-handed compression technique compresses the duct of interest between the non-scanning hand and the probe that is slid distally to include the nipple.
- The rolled nipple technique (Figure 12)
  ○ Depicts the portion of the mammary duct within the nipple
  ○ The probe rolls the nipple towards the finger of the contralateral hand.
- Ballottement maneuver:
  ○ Helps to visualize whether the material contained in the ducts can be mobilized and is more suggestive of layering debris than of solid content [18]. With Doppler, the echogenic secretions show more color with compression due to the movement.

## 4. US and Mammogram/MRI Correlation of Retroareolar Masses

**Figure 13:**
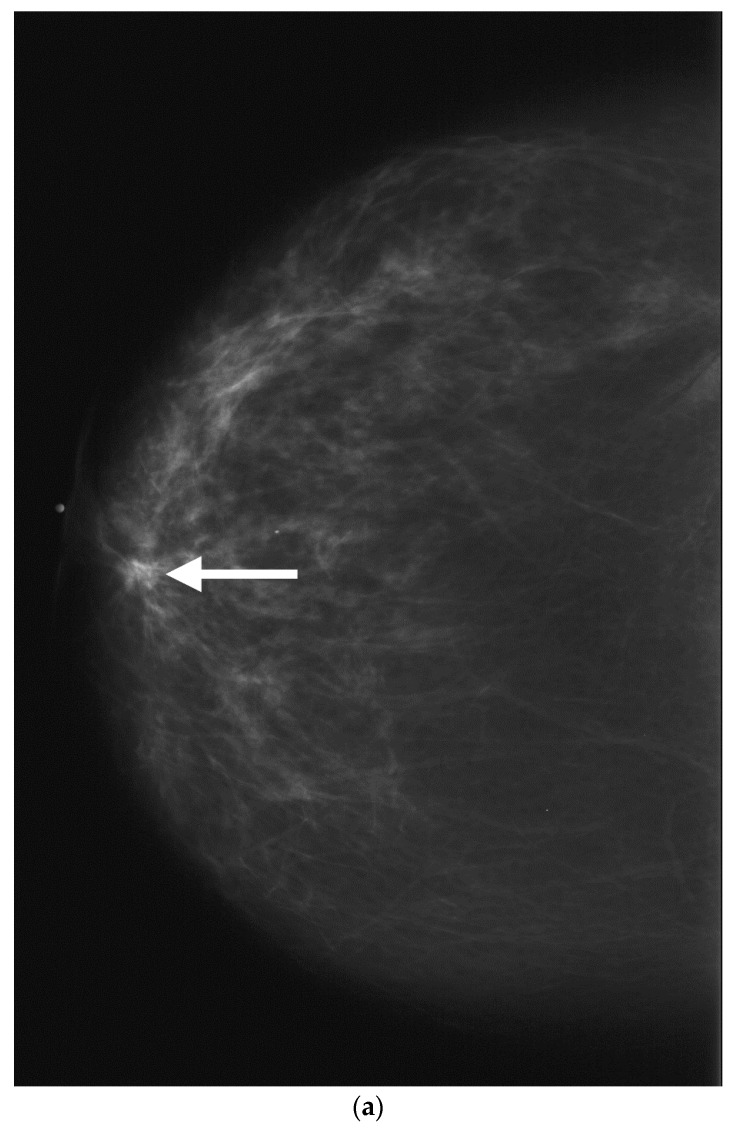

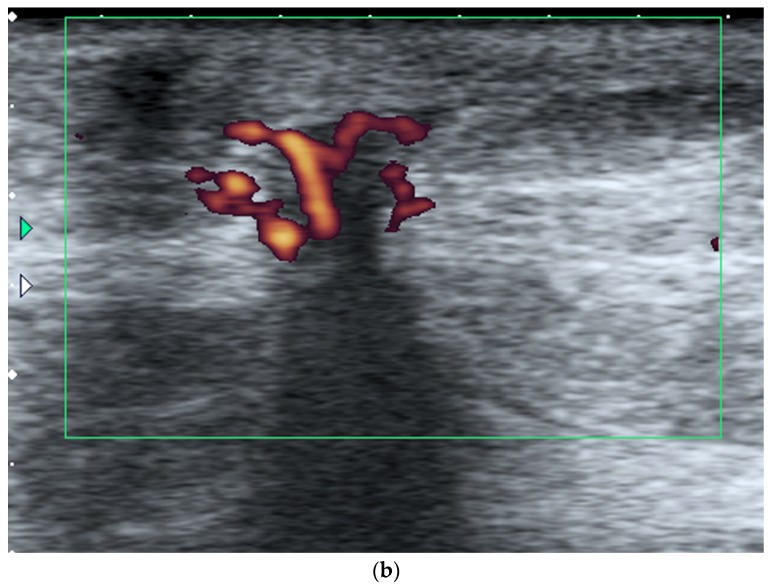
Screening routine right CC mammogram in a 60 year-old patient (**a**). The right CC mammogram demonstrates a small irregular spiculated mass in the retroareolar area (arrow). US images of the right breast show an (**b**); irregular spiculated non-parallel hypoechoic mass associated with an increased vascularity at color Doppler interrogation. Pathology yielded low-grade invasive ductal carcinoma.

**Figure 14:**
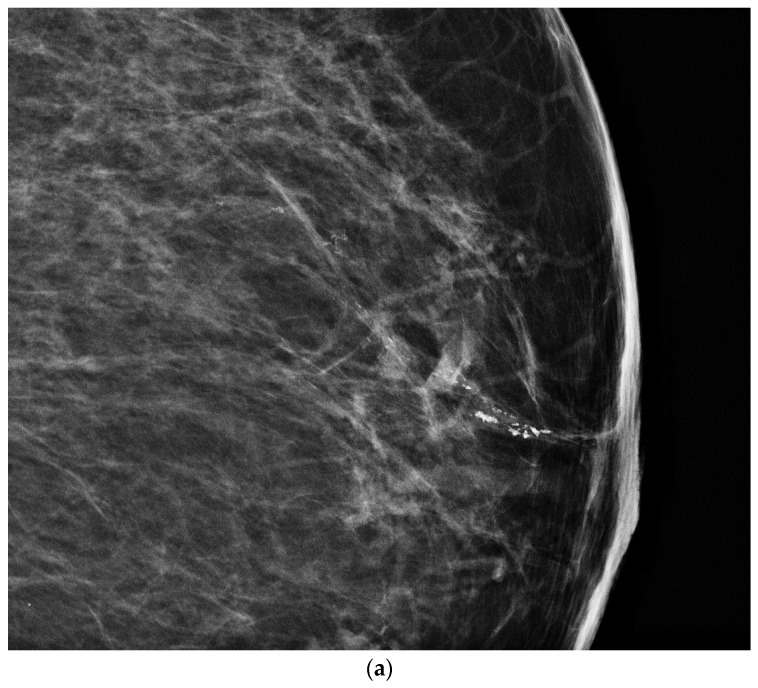

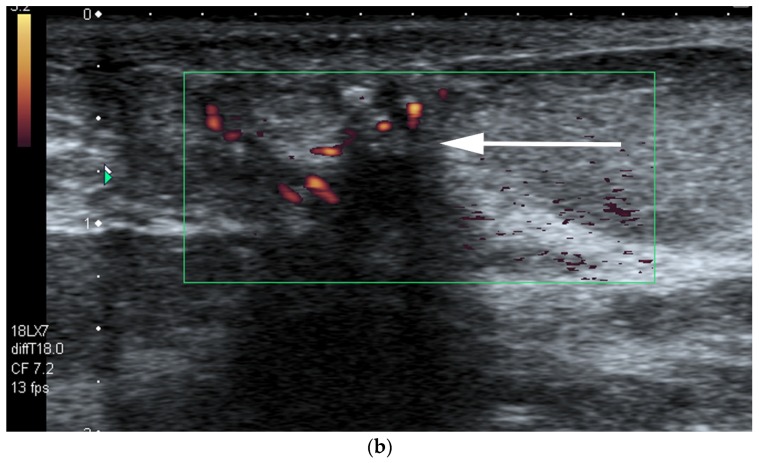
70 year-old patient referred for abnormal screening mammograms. Left CC magnification view demonstrates linear pleomorphic calcifications in a retroareolar location. US images shows microcalcifications are located within a dilated duct and associated with a rim vascularity at color Doppler interrogation (**b**) (arrow).

The new edition of the BI-RADS suggests combining the mammographic and US results performed the same day in a single report [18]. With a single interpretation, the radiologist integrates different findings into a unique conclusion (Figure 13 and Figure 14).

**Figure 15:**
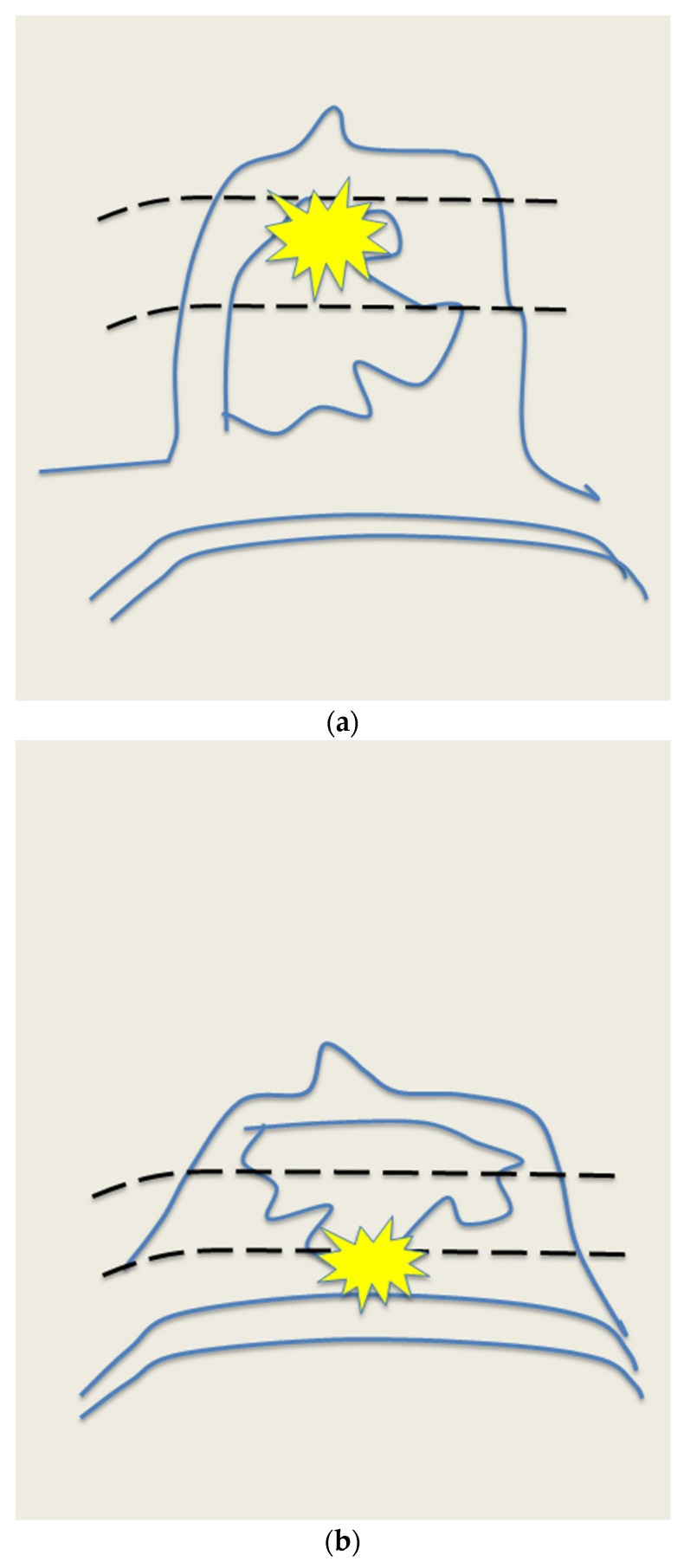
Example of position varying between mammogram (**a**) and US (**b**).

For breast US of the retroareolar region, as for any other location, the breast radiologist should correlate US findings with the mammographic or MRI findings in term of size, shape, location and surrounding breast tissue composition [19] (Figure 15). Positioning of the breast varies from one imaging modality to another, which might challenge mammographic/MRI/US correlations. However, in cases of retroareolar lesion, one can expect that the variation in location between US and other modalities (MRI and mammogram) would be less noticeable. Indeed, it has been shown that the more reliable measurement for second look ultrasound performed after breast MRI is the distance between the lesion and the nipple (reproducible between MRI and US) [20,21]. Therefore if the lesion is close to the nipple, the distance between the lesion and the nipple should not vary between MRI and US [20].

The new BI-RADS lexicon now introduces the new measurement in cm between a lesion and the nipple in addition to the quadrant location, depth and clock wise location. It also emphasizes the discrepancy of the nipple-lesion distance between mammograms and US [5,6,7,8,9,10,11,12,13,14,15,16,17,18,19,20,21,22]. Indeed, US is performed in a supine position while mammogram is performed in an upright position. As a consequence, the distance measured on mammogram and US can be discordant in some cases.

When clinical findings are worrisome and imaging is negative with normal standard dual-view mammogram, an additional US is suggested to exclude a pathologic process. In the same vein, a negative ultrasound cannot normalize an abnormal mammogram or suspicious clinical finding.

## 5. Role of the Retroareolar US in Clinical Practice

The latest edition of the BI-RADS lexicon, based on the ACRIN6666 study suggests that any breast imager (technologist and radiologist) should record one image of each quadrant plus one image behind the nipple [18,22]. This new statement emphasizes the importance in imaging the retroareolar region with particular attention, to the appropriate settings [18].

The fact that the number of incidentally detected carcinoma by US in the retroareolar region is significantly smaller than by other modalities (clinical, mammograms) supports the necessity for paying attention to the retroareolar location when performing routine US in clinical practice. 

## 6. Conclusions

With awareness of the complexity of the region, and knowledge of specific manoeuvers when doing breast US and appropriate settings to be used, breast imagers would be able to more appropriately scan the retroareolar regions and detect earlier stage breast cancers.
